# Modeling the effect of comprehensive interventions on Ebola virus transmission

**DOI:** 10.1038/srep15818

**Published:** 2015-10-30

**Authors:** Mingwang Shen, Yanni Xiao, Libin Rong

**Affiliations:** 1 School of Mathematics and Statistics, Xi’an Jiaotong University, Xi’an 710049, PR China; 2 Department of Mathematics and Statistics, Oakland University, Rochester, Michigan 48309, USA

## Abstract

Since the re-emergence of Ebola in West Africa in 2014, comprehensive and stringent interventions have been implemented to decelerate the spread of the disease. The effectiveness of interventions still remains unclear. In this paper, we develop an epidemiological model that includes various controlling measures to systematically evaluate their effects on the disease transmission dynamics. By fitting the model to reported cumulative cases and deaths in Guinea, Sierra Leone and Liberia until March 22, 2015, we estimate the basic reproduction number in these countries as 1.2552, 1.6093 and 1.7994, respectively. Model analysis shows that there exists a threshold of the effectiveness of isolation, below which increasing the fraction of latent individuals diagnosed prior to symptoms onset or shortening the duration between symptoms onset and isolation may lead to more Ebola infection. This challenges an existing view. Media coverage plays a substantial role in reducing the final epidemic size. The response to reported cumulative infected cases and deaths may have a different effect on the epidemic spread in different countries. Among all the interventions, we find that shortening the duration between death and burial and improving the effectiveness of isolation are two effective interventions for controlling the outbreak of Ebola virus infection.

The Ebola virus disease (EVD) affecting multiple countries in West Africa in 2014 has an unprecedented magnitude, which is far larger than all previous EVD outbreaks combined. By March 22, 2015, a total of 24,872 cases (including 14,682 confirmed, 3,564 probable, and 7,626 suspected cases) of EVD, as well as 10,311 deaths from the infection, have been reported in Guinea, Sierra Leone, Liberia^1^. Ebola virus spreads primarily via contact with body fluids of infectious people, and with those dead but not buried who can still transmit the virus in traditional West African funeral practices. Because of lack of licensed therapeutic treatment regimens and vaccines^2^, major control measures are a combination of early diagnosis, case isolation, contact precaution, awareness campaigns, and sanitary burial practices. Identifying infected people quickly by the polymerase chain reaction (PCR) assay and isolating them to break chains of EVD transmission may be effective to control the outbreak. The effect of reducing the time between symptoms onset and diagnosis with rapid testing has also been investigated in the literature^3^,^4^,^5^.

Chowell *et al*.^3^ used a mathematical model to study the effect of early diagnosis of pre-symptomatic individuals and found that the basic reproduction number always decreases as the fraction of early diagnosed individuals increases. The result may be affected by the effectiveness of isolation. Although the isolated class has a lower transmission rate than the non-isolated class, isolated individuals may live longer due to supportive clinical care and have more chance to infect others. Whether the relationship between the fraction of early isolation and the effectiveness of isolation affects the Ebola epidemic has not been explored. In this paper, we will use a model to systematically evaluate the effect of a number of factors, including isolation of pre-symptomatic individuals and the time between symptoms onset and isolation, on both the basic reproduction number and the final epidemic size in the above three African countries.

Media report on the numbers of infected cases and deaths can greatly affect social behavior and play an important role in defining health issues in the EVD epidemic^6^,^7^,^8^,^9^. Mathematical models have been used to explore the effect of mass media on infectious disease outbreaks such as SARS in 2003^10^,^11^,^12^ and H1N1 in 2009^13^,^14^,^15^,^16^,^17^, assuming that media coverage depends on the number of infected individuals and reduces the incidence rate. Very few models have considered the impact of media coverage on the transmission dynamics of EVD.

Recently, the first large-scale trials that aim to assess the safety and efficacy of two Ebola vaccines (cAd3-EBOZ and VSV-ZEBOV) were initiated in Liberia on February 2, 2015^18^. The effectiveness of these vaccines still remain unclear. We will include the effect of vaccination in our model. The objective of this paper is to assess the effect of all possible intervention strategies (isolation, media impact, safe burial, and vaccination) on controlling the spread of Ebola virus in Guinea, Sierra Leone, Liberia. We develop a mathematical model on the basis of the model in ref. 3 and fit to epidemiological data of reported cumulative numbers of infected cases and deaths^1^. Using the model, we evaluate the potential effect of increasing the fraction of latent individuals prior to symptoms onset, shortening the duration between symptoms onset and isolation, improving media coverage, following restrict burial procedures, and administrating timely vaccine on the epidemic of Ebola infection.

## Methods

### Model formulation

We develop a mathematical model (Eq. 1) to study the impact of comprehensive interventions including isolation, media impact, safe burial and vaccination. The population is divided into eight classes: *S* (susceptible individuals who can be infected by Ebola virus following a contact with infectious cases), *V* (vaccinated individuals), *E*_1_ (latent undetectable individuals), *E*_2_ (latent detectable individuals), *I* (infectious individuals with symptoms), *J* (isolated individuals), *D* (individuals who are dead but have not been buried; they can still transmit the disease during funerals), and *R* (recovered individuals). Let *N* denote the total population size, i.e., *N* = *S* + *V* + *E*_1_ + *E*_2_ + *I* + *J* + *R*.

Susceptible individuals become infected through contact with infectious individuals and enter into the latent class at rate 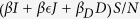, where *β* is the mean human-to-human transmission rate per day, 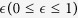 quantifies the relative transmissibility of isolated individuals compared to infectious symptomatic patients who are not isolated. Therefore, 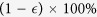 (reduction in transmissibility) provides a measure of the effectiveness of isolation. *β*_*D*_ is the transmission rate during funerals. In the model, 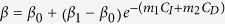, 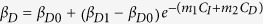, where *C*_*I*_ and *C*_*D*_ are the cumulative infected cases and deaths, respectively. *m*_1_ and *m*_2_ are non-negative parameters that measure the effect of media reported cumulative numbers of infected cases and deaths on the contact transmission. When *m*_*i*_ (*i* = 1, 2) is 0 or relatively small, the transmission rate *β* is equal or close to the constant *β*_1_. For *m*_*i*_ > 0, the public is more aware of the disease so that the transmission rate could be decreased to *β*_0_ (<*β*_1_) as the number of accumulated infected cases *C*_*I*_ or deaths *C*_*D*_ increases. Similarly, the transmission rate *β*_*D*0_ is assumed to be less than *β*_*D*1_ during funerals.

**Figure 1:**
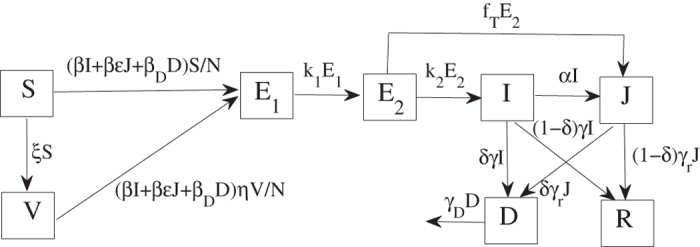
A schematic flow diagram of Ebola infection with isolation, media impact, post-death transmission and vaccination. The description of parameters can be found in Table 1.

We assume that susceptible individuals receive the vaccine at a rate *ξ*. The effectiveness of immunization is assumed to be *η* where 0 ≤ *η* ≤ 1 with *η* = 0 meaning that the vaccine is perfectly effective and *η* = 1 meaning that the vaccine has no effect. Latent undetectable individuals (*E*_1_) progress to the latent detectable class *E*_2_ at a rate *k*_1_, and subsequently move to the infectious symptomatic class *I* at a rate *k*_2_. Let *f*_*T*_ be the rate at which latent detectable individuals are detected and isolated. Thus, *θ* = *f*_*T*_/(*f*_*T*_ + *k*_2_) represents the fraction of isolated patients among latent detectable individuals exiting the class. Infectious individuals are isolated at a rate *α*, or removed from the class at a rate *γ* due to recovery (with probability 1 − *δ*) or disease-induced death (with probability *δ*). Similarly, isolated individuals are removed at a rate *γ*_*r*_ because of recovery (with probability 1 − *δ*) or disease-induced death (with probability *δ*). A flow diagram of the model (Eq. 1) is shown in Fig. 1 and parameters are described in Table 1. The dynamical model is as follows:


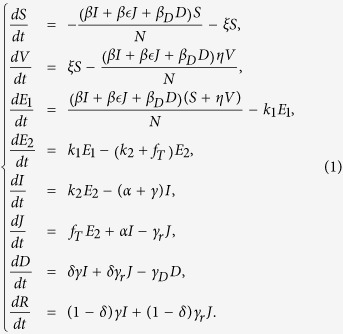


It should be noted that we keep track of the cumulative number of infected cases *C*_*I*_ and deaths *C*_*D*_ (*C*_*I*_ and *C*_*D*_ are not epidemiological states) by applying the solutions of Eq. (1) to the following equations


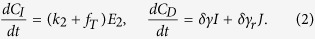


In the absence of effective vaccine (i.e. *ξ* = *η* = 0), we obtain the basic reproduction number *R*_0_ which is the spectral radius of the matrix 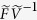 by using the next generation matrix approach given in^19^


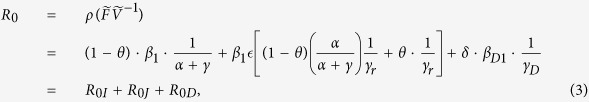


where *ρ* denotes the spectral radius and the matrices 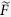 and 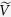 are


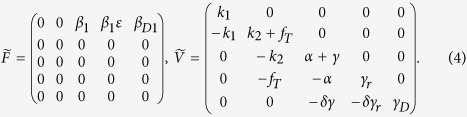


Note that each term of the aforementioned expression for *R*_0_ has clear epidemiological interpretation. 1 − *θ* = *k*_2_/(*f*_*T*_ + *k*_2_) is the fraction of latent detectable individuals becoming symptomatic among those exiting the *E*_2_ class. 1/(*α* + *γ*) is the mean infectious period of symptomatic cases (not isolated). *α*/(*α* + *γ*) is the fraction of symptomatic cases that are isolated among those exiting the *I* class. 1/*γ*_*r*_ is the mean infectious period of isolated cases and 1/*γ*_*D*_ is the mean duration of the infectious period between death and burial. *R*_0*I*_, *R*_0*J*_ and *R*_0*D*_ reflect the contribution to new infection from the symptomatic class (*I*), the isolated class (*J*), and the dead class (*D*), respectively. When vaccine is included, i.e., *ξ* > 0, *η* > 0, the reproduction number can be calculated as *R*_0*V*_ = *ηR*_0_, where *R*_0_ is given by (3).

### Data fitting and parameter estimation

We obtained data of the total cumulative cases and deaths (as the sum of confirmed, probable and suspected cases) for Guinea, Sierra Leone, Liberia from the World Health Organization (WHO)^1^. We used *t*_0_ (see Table 1) as the starting date for each country when the first infectious case was reported^20^. We compiled publicly available time series of reported cases and deaths from WHO beginning from 22 March 2014, 27 May 2014, and 17 June 2014 for Guinea, Sierra Leone, Liberia, respectively^20^,^21^. The data as of 22 March 2015 were used to fit the model equation (2) to estimate the unknown parameters. The latest data from 29 March 2015 to 3 May 2015 were used to assess how well our forecasts match additional data points.

Based on previous studies, the mean incubation time for EVD is 7 days^3^ (1/*k*_1_ + 1/*k*_2_ = 7) with 4 days from the latent undetectable class to latent detectable class (1/*k*_1_ = 4) and 3 days from the latent detectable class to symptomatic class (1/*k*_2_ = 3)^3^,^22^. The mean time from symptoms onset to isolation is 3 days (1/*α* = 3). The mean time that infectious individuals are removed from the class after recovery or disease-induced death is 6 days (1/*γ* = 6)^3^,^5^. The average duration from death to burial is chosen as 2 days (1/*γ*_*D*_ = 2)^23^. As for the total population size *N*, we used the number of 11745189 for Guinea, 6092075 for Sierra Leone, and 4294077 for Liberia, provided by the World Bank^24^. The other parameters were estimated by fitting the equation (2) to the data on cumulative cases and deaths of each country and using an adaptive Metropolis-Hastings (M-H) algorithm to carry out the Bayesian Markov Chain Monte Carlo (MCMC) procedure implemented by *Matlab*. The estimated parameter values and their 95% confidence intervals are listed in Table 1.

## Results

### Model prediction and comparison with data

**Figure 2:**
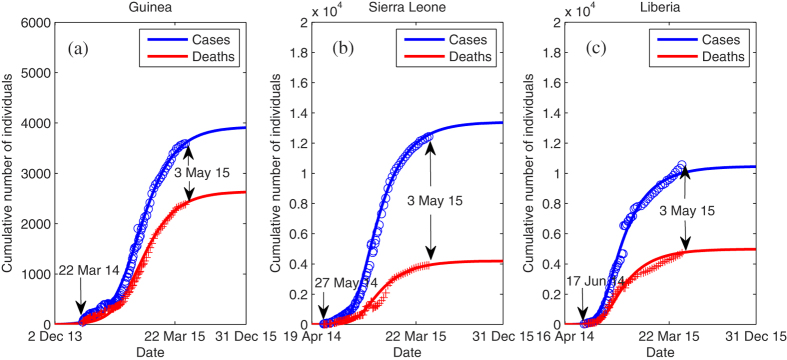
Model fit to the Ebola data in Guinea, Sierra Leone and Liberia. Data of the cumulative numbers of infected cases and deaths are shown as blue circles and red pluses, respectively. The solid line represents the best fit to the data.

We first consider the situation without vaccination because of the unavailability of vaccine before February 2, 2015, and then explore the effect of vaccination in Liberia where the first large-scale vaccine trials were implemented since then^18^. Figure 2 shows that the model provides a very good fit to the reported data of both infected cases and deaths in all three countries. Using our parameter estimates (Table 1), we calculated the basic reproduction number *R*_0_ in Guinea, Sierra Leone and Liberia as 1.2552, 1.6093 and 1.7994, respectively. They are all within the range of estimated values in the literature (see Table 2). In Guinea, the symptomatic component of *R*_0_ accounts for 0.1844 (14.7%), the isolated component for 0.7515 (59.9%) and the dead component for 0.3193 (25.4%). For the epidemic in Sierra Leone, transmission by the symptomatic, isolated and dead class accounts for 0.2668 (16.6%), 1.2375 (76.9%), and 0.1049 (6.5%), respectively, in the value of *R*_0_. For Liberia, these three components account for 0.2835 (15.8%), 1.2314 (68.4%), and 0.2846 (15.8%), respectively.

**Figure 3:**
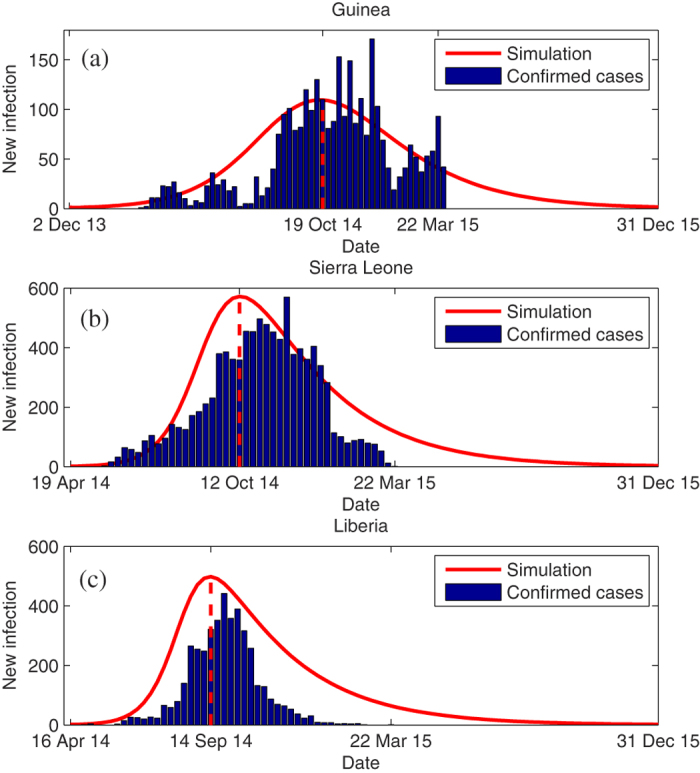
The estimated number of weekly total reported cases in the fitted model (red solid lines) and time-series data of the weekly number of confirmed cases (blue bars) reported by the WHO 1. The vertical red dash line indicates when the epidemic reached the peak.

We plotted the simulated newly infected individuals in Fig. 3 (red solid lines) and compared with the reported weekly confirmed cases in the three countries. Most of the reported cases in Guinea were confirmed (3011/3429), while only 8520/11841 and 3151/9602 (the numerator and denominator denote the confirmed and total cases on 22 March 2015, respectively) of cases were confirmed in Sierra Leone and Liberia, respectively. There is a small difference between the simulated new infection and reported data in Guinea (Fig. 3(a)), while the simulated results are higher than confirmed cases in Sierra Leone and Liberia (Fig. 3(b,c)) due to a lower confirmation rate in these two countries. Moreover, the simulated trend of EVD outbreak and predicted peak time are in good agreement with what the WHO data^1^ and other references^25^,^26^,^27^,^28^ indicated.

**Figure 4:**
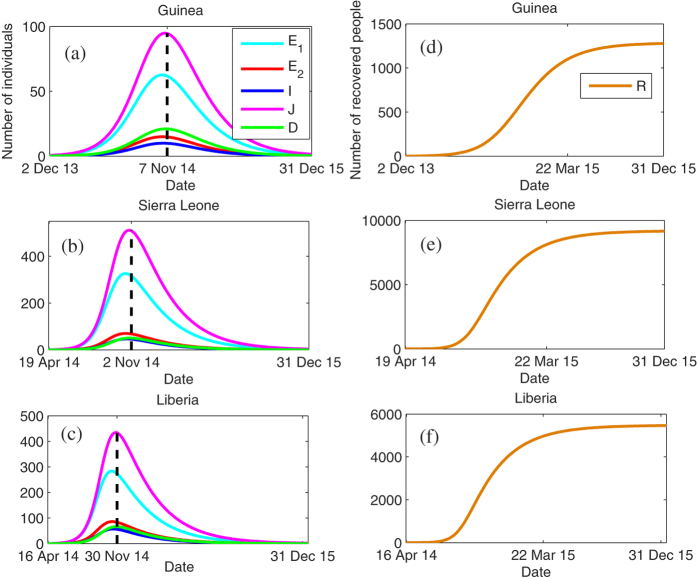
Time evolution of the latent undetectable (*E*_1_), latent detectable (*E*_2_), infectious symptomatic (*I*), isolated (*J*), dead but not buried (*D*), and recovered (*R*) classes. The vertical black dash line indicates the time when the total number of infected individuals reaches the peak.

The solution of model (1) is shown in Fig. 4. The number of infected individuals is decreasing and tends to vanish gradually at the end of 2015 (Fig. 4(a–c)). Figure 4(d–f) show that the number of simulated recovered individuals on 22 Mar 2015 is 1097 in Guinea, 8105 in Sierra Leone, and 5033 in Liberia. They are very close to the exact numbers (1166, 8094, and 5301, respectively) in the three countries.

#### Effect of isolation on *R*_0_ and the final epidemic size

To explore the effect of early and broad diagnosis on the basic reproduction number *R*_0_, we calculate the derivative of *R*_0_ with respect to the parameter *θ* (i.e. the fraction of latent individuals diagnosed prior to symptoms onset) as follows


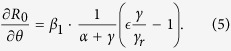


**Figure 5:**
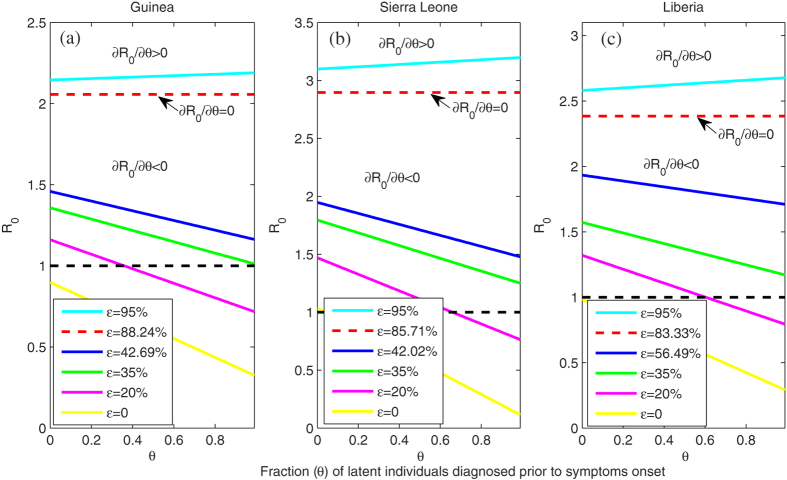
The effect of early diagnosis of pre-symptomatic individuals on the basic reproduction number *R*_0_ when the relative transmissibility *ϵ* of isolated classes is varied. All the other parameters are listed in Table 1.

The derivative is greater than 0 when 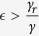 and less than 0 when 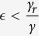. Thus, when the effectiveness of isolation 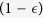 is low (i.e. 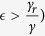, increasing the fraction *θ* of detection of pre-symptomatic cases can increase the basic reproduction number *R*_0_. This is harmful to the control of the disease. If 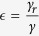, increasing or decreasing *θ* does not have any effect on *R*_0_. Only when isolation is effective (i.e. 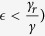, increasing the fraction *θ* of pre-symptomatic patients to be detected is beneficial to disease control. In this paper, we have the threshold 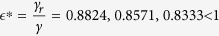 for Guinea, Sierra Leone and Liberia respectively (see Table 1), i.e., 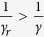, indicating that the infectious period of isolated individuals in these three countries are longer than that for non-isolated individuals. The relative transmissibility 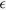 of isolated classes in Guinea, Sierra Leone and Liberia are estimated as 0.4269, 0.4202, and 0.5649 (see blue lines in Fig. 5), respectively, which are all less than their respective threshold. Thus, increasing the fraction *θ* of detecting pre-symptomatic cases results in a decline in the basic reproduction number. If the effectiveness of isolation increases to 80%, i.e., the relative transmissibility 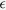 of isolated individuals decreases to 20% (magenta lines in Fig. 5), then about 35%, 65%, 60% of pre-symptomatic patients need to be detected in Guinea, Sierra Leone and Liberia to control the disease. If the isolation is perfect 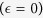, then the disease could be controlled easily (see yellow lines in Fig. 5).

**Figure 6:**
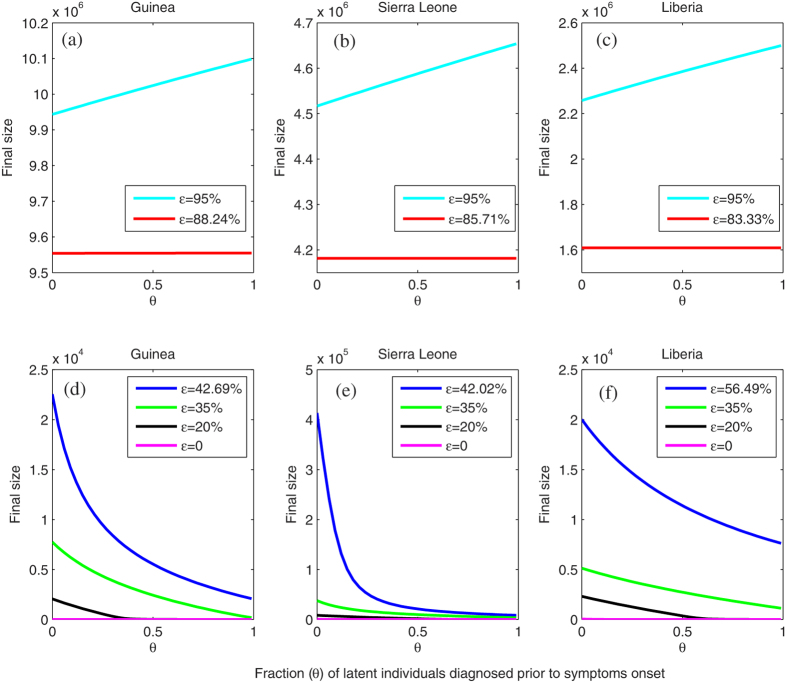
The effect of early diagnosis of pre-symptomatic individuals on the final epidemic size when the relative transmissibility *ϵ* of isolated classes is varied. All the other parameters are listed in Table 1.

Next, we study the effect of the fraction *θ* of latent individuals diagnosed prior to symptoms onset on the final epidemic size, which is defined as *Z* = *C*_*I*_(*t*_1_) = *C*_*D*_(*t*_1_) + *R*(*t*_1_)^29^. The time *t*_1_ is *min*{*t* > 0,*E*_1_(*t*) + *E*_2_(*t*) + *I*(*t*) + *J*(*t*) + *D*(*t*) = 0}. Although the explicit formula between the final size *Z* and *θ* cannot be obtained, there exists a threshold which decides whether the final epidemic size increases as *θ* increases. The relationship between *Z* and *θ* is similar to that between *R*_0_ and *θ*. For example, it follows from Fig. 6(a) that if the effectiveness of isolation is lower than 11.76%, i.e., the relative transmissibility 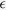 of isolated class is greater than 88.24%, then the final epidemic size increases as *θ* increases. This suggests that strict measures must be taken to limit the contact of isolated individuals. Otherwise, low effectiveness of isolation would lead to more infected individuals because isolated people live longer due to improved medical care and have more chances to infect other people. The benefit of having a reduced transmission rate would be counteracted by a longer infectious period of the isolated class. Fig. 6(d–f) show the situation in which the final epidemic size decreases as *θ* increases.

Using the estimated value of *θ* (see Table 1, *θ* = 0.6816,0.7132,0.5950 in Guinea, Sierra Leone and Liberia, respectively), the simulated final size is 3907, 13360 and 10439 in these three countries respectively (blue lines in Fig. 6). This simulated result in Liberia where the outbreak of EVD was considered ended by the WHO^30^ on 9 May 2015 is very close to the actual final size 10564 with a relative error 1.18%.

**Figure 7:**
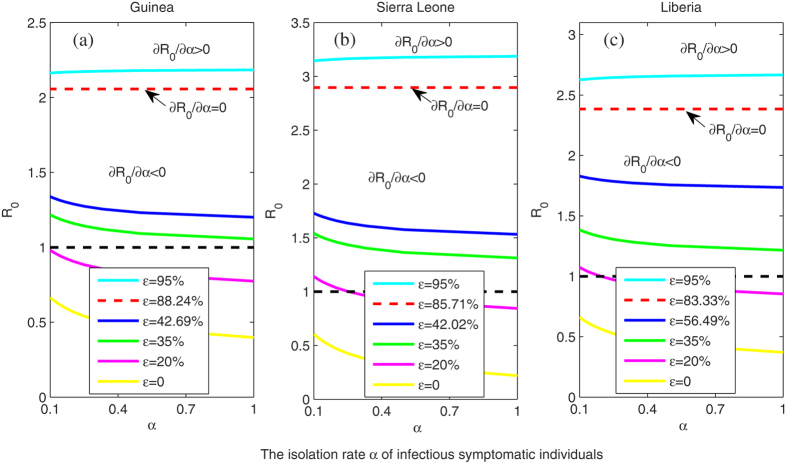
The effect of isolation rate *α* of infectious symptomatic individuals on the basic reproduction number *R0* when the relative transmissibility *ϵ* of isolated classes is varied. 1/*α* is the time between symptom onset and isolation. All the other parameters are listed in Table 1.

Similarly, to investigate the effect of shortening the time (1/*α*) between symptom onset and isolation (i.e, increasing the isolation rate *α* of infectious symptomatic individuals) on the basic reproduction number *R*_0_, we plot Fig. 7, which shows a similar relationship to that between *R*_0_ and *θ* when the the relative transmissibility 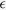 of isolated class varies. This is not surprising because the derivative of *R*_0_ with respect to *α* is


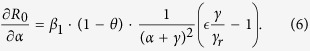


It has the same threshold 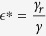 that decides whether the basic reproduction number increases with an increasing *α*. If the relative transmissibility 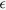 of isolated individuals decreases to 20% (see magenta lines in Fig. 7), then the disease will be under control if the isolation rate *α* exceeds 0.2 (i.e., the time between symptom onset and isolation is less than 5 days) in Sierra Leone and Liberia. In this case 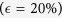, EVD will always be controlled no matter how long the time (1/*α*) is in Guinea.

### Effect of media coverage on the final epidemic size

**Figure 8:**
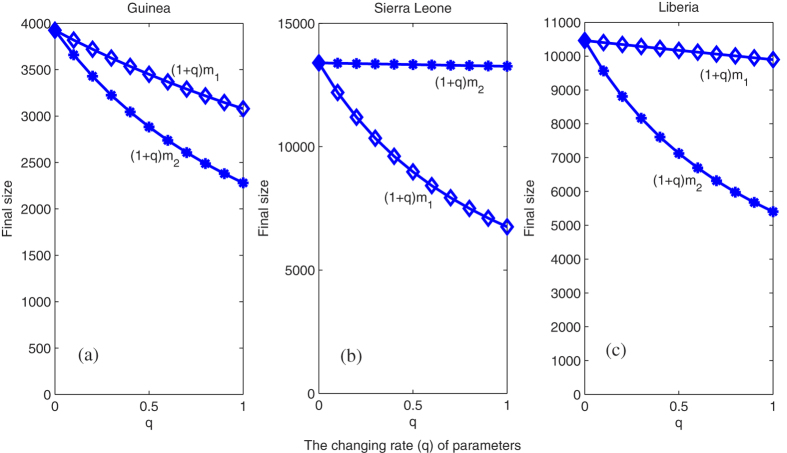
Change of the final epidemic size in Guinea, Sierra Leone and Liberia with different values of *q* (the percentage of increase of the media impact coefficients *m*_1_ and *m*_2_). All the other parameters are listed in Table 1.

We study the effect of media coverage *m*_1_ (response to the reported cumulative number of infected cases) and *m*_2_ (response to the reported cumulative deaths) on the final epidemic size. Let *q* (0 ≤ *q* ≤ 1) be the percentage of increase of *m*_1_ and *m*_2_ from its baseline estimate (Table 1). We examine how the final epidemic size changes with an increasing media impact *q* in Fig. 8. For example, increasing *m*_1_ (or *m*_2_) by 30% from its baseline value (while keeping other parameters fixed) can reduce the final epidemic size by 7.62% (or 17.81%) in Guinea, 22.79% (or 0.31%) in Sierra Leone, and 1.68% (or 21.95%) in Liberia, respectively. This indicates that the response to the reported cumulative deaths (*m*_2_) may be stronger than the response to reported cumulative cases (*m*_1_) in Guinea and Liberia. A possible explanation of this result is that the case fatality rate in these two countries is relative high (see Table 1). Thus, people had increased attention to EVD-induced deaths. In Sierra Leone, an opposite scenario was observed. In conclusion, the analysis of media impact indicates that massive news coverage on cumulative cases and deaths would greatly curb the spread of the Ebola disease.

### Effect of post-death transmission on *R*_0_

**Figure 9:**
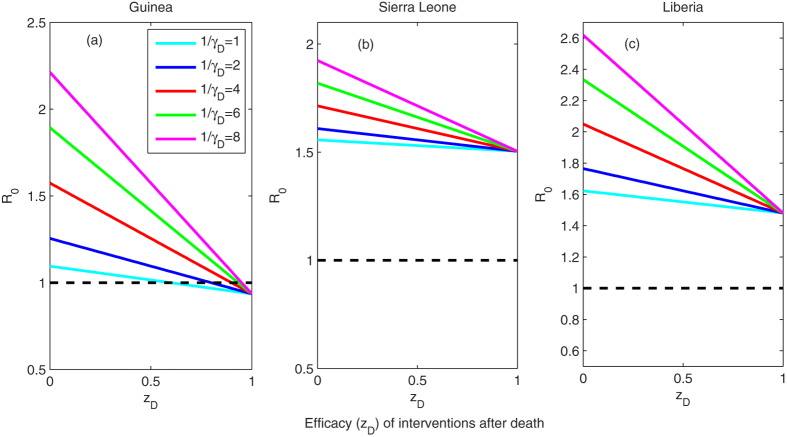
The effect of controlling post-death transmission of EVD on the basic reproduction number *R*_0_. *z*_*D*_ is the efficacy of intervention at funerals. The infection rate *β*_D_ becomes *β*_D_(1 *−* *z*_D_) after intervention. The duration 1*/γ*_D_ of traditional burials changes from 1 to 8 days. All the other parameters are listed in Table 1.

To assess the effect of reducing post-death transmission on the basic reproduction number *R*_0_, we plot the variation in *R*_0_ with the efficacy of intervention, *z*_*D*_, at funerals (i.e., the post-death transmission rate *β*_*D*_ is decreased to *β*_*D*_(1 − *z*_*D*_)) for various durations of the traditional burial 1/*γ*_*D*_ in Fig. 9. It shows that increasing the efficacy of intervention leads to a decline in *R*_0_ in all three countries. In particular, the epidemic in Guinea could be controlled by following very restrict burial procedures (a large *z*_*D*_). For example, when 1/*γ*_*D*_ = 2 (blue line in Fig. 9(a)) and the efficacy of interventions *z*_*D*_ exceeds about 70%, the basic reproduction number *R*_0_ will become less than 1. However, reducing transmission during funerals is insufficient to control the disease in Sierra Leone or Liberia no matter how large *z*_*D*_ is (Fig. 9(b–c)). This is because the burial-related transmission contributes less to the spread of the disease in Sierra Leone (*R*_0*D*_/*R*_0_ = 6.5%) and Liberia (*R*_0*D*_/*R*_0_ = 15.8%) than in Guinea (*R*_0*D*_/*R*_0_ = 25.4%). Figure 9 also demonstrates that *R*_0_ decreases as the duration of funeral decreases. This suggests that the duration of funeral should be as short as possible for the control of the disease.

### Sensitivity analysis

**Figure 10:**
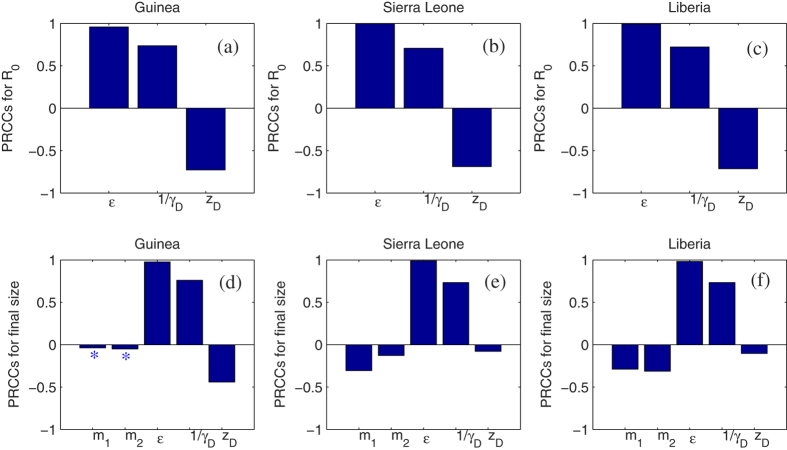
Sensitivity test of the basic reproduction number and the final epidemic size on parameters. (**a**–**c**): PRCCs for *R*_0_. (**d**–**f**): PRCCs for the final epidemic size. The sample size is set to 1000. The star (*) in Fig. (d) means that PRCCs are not significant (p-value > 0.01).

In order to identify which parameters the basic reproductive number *R*_0_ and the final epidemic size are most sensitive to, we perform sensitivity analysis using the Latin Hypercube Sampling (LHS) technique and calculate the partial rank correlation coefficients (PRCCs)^31^,^32^ in Fig. 10. The magnitude of these PRCCs shows the sensitivity of these parameters and the sign of the PRCCs indicates a positive or negative correlation between the inputs (i.e. parameters) and outputs (i.e. *R*_0_ and the final epidemic size). It follows from Fig. 10(a–c) that the two parameters with the most significant impact on *R*_0_ are the relative transmissibility 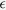 of isolated classes and duration of the burial 1/*γ*_*D*_. A smaller relative transmissibility 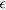 of isolated classes or a shorter duration of burial results in a smaller *R*_0_, which is consistent with the results shown in Figs 5 and 9. These two parameters 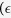 and 1/*γ*_*D*_) also have the most significant impact on the final epidemic size, as shown in Fig. 10(d–f). These results suggest that increasing the effectiveness of isolation (decreasing 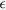) and shortening the duration of funerals (1/*γ*_*D*_) are of crucial importance to reduce the EVD infection. The effect of media (*m*_1_ or *m*_2_) has no effect on *R*_0_ (see the formula of *R*_0_ in (3)), which is in agreement with the findings in refs. 10, 11, 12,17 that the media impact does not affect the epidemic threshold. However, the media impact leads to a decline in the final epidemic size, as shown in Fig. 8.

### Effect of vaccination in Liberia

**Figure 11:**
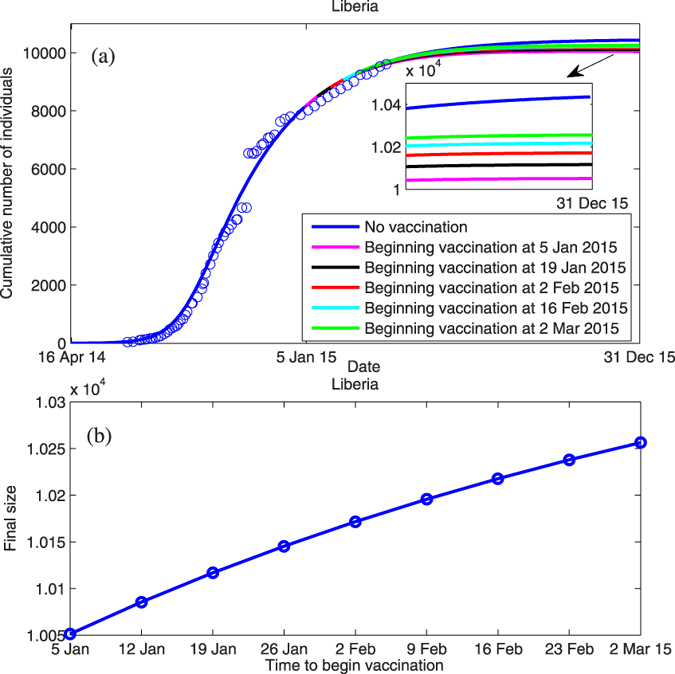
Projected impact of different vaccination timing on the cumulative cases and the final epidemic size in Liberia.

Effective vaccination, if used before the epidemic peaks, would be projected to prevent tens of thousands of deaths. In Liberia, the EVD epidemic reached the peak around mid-September 2014^26^,^27^, which is in good accordance with our simulated peak time, 14 Sep 2014, as shown in Fig. 3(c). Large-scale trials of vaccination were initiated on 2 February 2015 in Liberia but the efficacy of vaccination is unclear. To understand the effect of the timing of vaccine administration on the spread of EVD and the final epidemic size, we fit the model (1) to the cumulative infected cases in Liberia from 2 February 2015 to 22 March 2015 and estimated the vaccination rate *ξ* and the efficacy of vaccine *η* (see Table 1). After initiating the vaccine, the reproduction number is reduced from 1.7994 to 0.9873 (Table 1). Using the estimated values of *ξ* and *η*, we plotted how the simulated cumulative cases would vary with different timing of vaccination. It shows that the earlier the vaccination is initiated, the lower the cumulative cases (Fig. 11(a)). It follows from Fig. 11(b) that the final epidemic size grows with delayed vaccination. There would be 20 more cases if vaccination had started one week later (Fig. 11(b)). This indicates that the timing of vaccine administration does not have a big effect on the final size when the epidemic declines.

## Conclusion and Discussion

In this study, we developed a mathematical model to study the transmission dynamics of Ebola virus. The model includes the effect of case isolation, media impact, post-death transmission and vaccination. By fitting the model to the WHO reported data of infected cases and deaths (Fig. 2), we obtained reasonable estimates of the parameters (Table 1). The basic reproduction number in Guinea, Sierra Leone and Liberia is estimated as 1.2552, 1.6093 and 1.7994, respectively. They are all in good agreement with previous estimates (Table 2). The simulated results indicate that the outbreak in the above countries reaches the peak on 19 Oct 2014, 12 Oct 2014, 14 Sep 2014, respectively (see Fig. 3), which also agrees with the observations in Sierra Leone^25^,^27^,^28^ and in Liberia^26^,^27^,^28^.

We found that isolation does not always contribute to the control of the EVD transmission. Whether this intervention is beneficial depends on the effectiveness of isolation (or the relative transmissibility 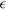 of isolated individuals). If the infectious period of isolated individuals is less than that of non-isolated, i.e., 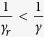 or 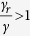, then isolation is always beneficial to disease control because of a lower transmission rate and a shorter infectious period of isolated individuals. On the contrary, if the infectious period of isolated individuals is longer than that of non-isolated, then when the isolation effectiveness is relatively low (e.g. 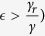, there will be more infected cases as the fraction *θ* of latent individuals prior to symptoms onset increases (Figs 5 and 6(a–c)) or the duration 1/*α* between symptoms onset and isolation decreases (Fig. 7). When the isolation effectiveness is high (e.g. 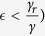, then enhancing isolation can greatly reduce new infection (Figs 5, 6(d–f), and 7). These results suggest that isolation may not always have a positive effect on disease control as shown in refs. 3, 4, 5. Sensitivity analysis also shows that the relative transmissibility 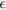 of isolated classes has the most significant impact on both the basic reproduction number and the final epidemic size, which further explains why the isolation effectiveness determines whether the disease can be controlled successfully. Therefore, strict measures should be taken to limit the contact with isolated individuals.

A few reasons may explain why the isolated class contributes more to the basic reproductive number *R*_0_ than the symptomatic class and the dead class. One is that the infectious period of isolated individuals (i.e. 1/*γ*_*r*_) is longer than that for non-isolated individuals because of improved clinical care. This is consistent with the parameter estimates shown in Table 1. The other reason is that a majority of patients (*θ*) are isolated but the isolation may not be effective in reducing the transmission of the disease. Ebola virus is very contagious and the transmission is also rapid, which makes isolation, as a containment strategy, usually inefficient^33^. This agrees with the estimate of 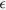 in Table 1, which is not small, implying that isolated individuals still have a high capacity to transmit the virus.

In our model, we did not discriminate isolated individuals from the latent detectable class (*E*_2_) and from the infectious class with symptoms (*I*). Isolated cases from the *E*_2_ class may have a different duration (1/*γ*_*r*_) staying in the isolated class from the isolated cases coming from the *I* class. However, the above conclusion that the isolated class contributes more to *R*_0_ should still be valid as long as the infectious period of isolated individuals is longer than that of their corresponding preceding class. This is usually true in view of the improved health care received for isolated individuals.

We also found that the estimate of the case fatality rate *δ* in Guinea (0.6728) is almost as twice as that in Sierra Leone (0.3143, see Table 1). One reason is a low level of preparedness, as well as poor availability and quality of medical care in Guinea^34^. Another important reason is that the outbreak in Guinea was caused by the Zaire strain^35^, which induces an average fatality rate of 79%, the highest death rate of the five known Ebola strains^34^.

Media impact can significantly affect the Ebola infection, as shown in Fig. 8. However, the responses to the reported cumulative deaths and cumulative cases may have different effect on the infection. We find that the response to the reported cumulative deaths (*m*_2_) is more sensitive than the response to the reported cumulative cases (*m*_1_) in Guinea and Liberia. It can be explained by a higher case fatality rate in these two countries (Table 1).

Post-death transmission is an important factor that induces more infection and hence can not be ignored during EVD outbreaks^23^,^36^. Our simulation shows that increasing the efficacy of intervention at funerals can control the disease in Guinea. However, this measure is insufficient to eliminate the disease in Sierra Leone and Liberia (shown in Fig. 9) because the burial-related transmission does not contribute much to the spread of the disease in these two countries. Sensitive analysis indicates that the duration of the burial 1/*γ*_*D*_ is the second parameter which most affects *R*_0_ and the final epidemic size. Thus, shortening the duration between death and burial and reducing transmission before burial would effectively reduce the infection.

Because the interventions we considered mainly address isolation of infected people within EVD treatment centres, one limitation of our model is that we do not explicitly account for hospital bed capacity, which plays an important role in affecting both the outbreak dynamics and the intervention efforts^37^. Additionally, the conventional homogeneous mixing assumptions used in our model may be over-simplified, especially in countries where infection always occurs in households due to the structure of the community^38^. Taking into consideration the effect of social networks structure on Ebola infection would also be interesting for future research.

In summary, we used an epidemiological model to study the effect of various measures on EVD transmission dynamics. Depending on the effectiveness of isolation, early and massive diagnosis of pre-symptomatic individuals with rapid testing may remain beneficial to reduce the transmission of the disease. Shortening the duration between death and burial and improving the effectiveness of isolation are two effective interventions for controlling the EVD outbreak.

## Additional Information

**How to cite this article**: Shen, M. *et al*. Modeling the effect of comprehensive interventions on Ebola virus transmission. *Sci. Rep*. **5**, 15818; doi: 10.1038/srep15818 (2015).

## Figures and Tables

**Table 1:** Parameters and values for simulation and data fitting

Parameter	Description	Default or estimated mean value with 95% confidence interval			Source
		Guinea	Sierra Leone	Liberia	
*N*	Size of the total population	11,745,189	6,092,075	4,294,077	24
1/*k*_1_	Mean time from latent undetectable class to latent detectable class	4 days	4 days	4 days	3,22
1/*k*_2_	Mean time from latent detectable class to infectious symptomatic class	3 days	3 days	3 days	3,22
1/*α*	Mean time from infectious symptomatic class to isolated class	3 days	3 days	3 days	3,5
1/*γ*	Mean time that infectious individuals are removed by recovery or disease-induced death	6 days	6 days	6 days	3,5
1/*γ*_*r*_	Mean time that isolated individuals are removed by recovery or disease-induced death	6.7981 days[6.5617,7.0522]	6.9979 days[6.6578,7.3746]	7.2000 days[7.0522,7.3529]	Fitted
1/*γ*_*D*_	Mean time from death to traditional burial	2 days	2 days	2 days	23
*β*_1_	Pre-media human-to-human transmission rate	0.2896 day^−1^[0.2487,0.3305]	0.4652 day^−1^[0.4056,0.5247]	0.3500 day^−1^[0.3371,0.3628]	Fitted
*β*_0_	Post-media human-to-human transmission rate	0.2275 day^−1^[0.2088,0.2462]	0.2355 day^−1^[0.2069,0.2641]	0.1701 day^−1^[0.1644,0.1758]	Fitted
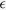	Relative transmissibility of isolated individuals	0.4269[0.3836,0.4703]	0.4202[0.3558,0.4846]	0.5649[0.5392,0.5907]	Fitted
*β*_*D*1_	Pre-media transmission rate during funeral	0.2373 day^−1^[0.1926,0.2820]	0.1669 day^−1^[0.1266,0.2071]	0.2986 day^−1^[0.2693,0.3279]	Fitted
*β*_*D*0_	Post-media transmission rate during funeral	0.0445 day^−1^[0.0347,0.0543]	0.1367 day^−1^[0.1283,0.1452]	0.1214 day^−1^[0.1054,0.1375]	Fitted
*m*_1_	Response to the reported cumulative number of infected cases	1.2748 × 10^−4^[1.1280,1.4217] × 10^−4^	3.2539 × 10^−4^[3.1081,3.3997] × 10^−4^	2.9495 × 10^−5^[2.9084,2.9906] × 10^−5^	Fitted
*m*_2_	Response to the reported cumulative deaths	5.2235 × 10^−4^[5.0956,5.3514] × 10^−4^	1.2086 × 10^−5^[1.1171,1.3000] × 10^−5^	1.2 × 10^−3^[1.1884,1.2116] × 10^−3^	Fitted
*δ*	The case fatality rate	0.6728[0.6573,0.6884]	0.3143[0.3014,0.3272]	0.4765[0.4738,0.4792]	Fitted
*f*_*T*_	The rate at which latent detectable individuals progress to the isolation class	0.7136 day^−1^[0.6238,0.8033]	0.8291 day^−1^[0.7509,0.9072]	0.4898 day^−1^[0.4636,0.5160]	Fitted
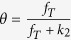	The fraction of isolated people among latent detectable individuals who exit this class	0.6816[0.6517,0.7067]	0.7132[0.6926,0.7313]	0.5950[0.5817,0.6075]	Calculated
*ξ*	The vaccination rate	——	——	1.3 × 10^−3^[0.7389,1.8611] × 10^−3^	Fitted
*η*	The efficacy of vaccination	——	——	0.5487[0.4649,0.6325]	Fitted
*R*_0*V*_	The reproduction number with vaccination	——	——	0.9873[0.8438,1.1308]	Calculated
*t*_0_	Date of the first reported infectious case	2 Dec 2013	19 Apr 2014	16 Apr 2014	20
*R*_0_	The basic reproduction number	1.2552[1.2211,1.2893]	1.6093[1.5609,1.6577]	1.7994[1.7655,1.8333]	Calculated
*R*_0*I*_	The symptomatic class's contribution to *R*_0_	0.1844[0.1636,0.2052]	0.2668[0.2440,0.2893]	0.2835[0.2698,0.2973]	Calculated
*R*_0*J*_	The isolated class's contribution to *R*_0_	0.7515[0.7032,0.7944]	1.2375[1.2026,1.2593]	1.2314[1.2144,1.2472]	Calculated
*R*_0*D*_	Contribution to *R*_0_ from post-death transmission	0.3193[0.2535,0.3857]	0.1049[0.0803,0.1293]	0.2846[0.2570,0.3121]	Calculated

**Table 2:** Published estimates of the basic reproduction number *R*_0_ for Ebola in Guinea, Sierra Leone, and Liberia.

Location	*R*_0_	95% CI (if given)	Reference
Guinea	1.11		21
	1.2552	[1.2211,1.2893]	Obtained here
	1.52		20
	1.71	[1.44,2.01]	39
	1.79	[1.47,1.79]	37
Sierra Leone	1.26		21
	1.32	[1.19,1.37]	37
	1.6093	[1.5609,1.6577]	Obtained here
	2.02	[1.79,2.26]	39
	2.42		20
Liberia	1.54		21
	1.63	[1.59,1.66]	40
	1.65		20
	1.73	[1.66,1.83]	41
	1.7994	[1.7655,1.8333]	Obtained here
	1.81	[1.34,2.75]	37
	1.83	[1.72,1.94]	39
	1.84	[1.60,2.13]	42
	2.49	[2.38,2.60]	43
